# Signal Transducer and Activator of Transcription 3 Control of Human T and B Cell Responses

**DOI:** 10.3389/fimmu.2018.00168

**Published:** 2018-02-07

**Authors:** Elissa K. Deenick, Simon J. Pelham, Alisa Kane, Cindy S. Ma

**Affiliations:** ^1^Immunology Division, Garvan Institute of Medical Research, Darlinghurst, NSW, Australia; ^2^St Vincent’s Clinical School, UNSW Sydney, Darlinghurst, NSW, Australia; ^3^Department of Immunology and Allergy, Liverpool Hospital, Liverpool, NSW, Australia; ^4^South Western Sydney Clinical School, UNSW Sydney, Liverpool, NSW, Australia

**Keywords:** signal transducer and activator of transcription 3, T cells, B cells, immunodeficiency, immune dysregulation

## Abstract

Signal transducer and activator of transcription 3 (STAT3) is a transcription factor that is activated downstream of many key cytokine receptors expressed by lymphocytes. As such, it plays a critical role in regulating B cells as well as CD4^+^ and CD8^+^ T cells. Patients with clinically significant immunodeficiency and immune dysregulation resulting from loss-of-function or gain-of-function mutations in STAT3 have been described. These individuals provide insight into the critical role of this transcription factor in the regulation of immune responses and the balance between effective immune protection and autoimmunity.

## Introduction

Signal transducer and activator of transcription 3 (STAT3) is a transcription factor that is activated downstream of a large range of cell surface receptors. It forms part of a family of proteins that also includes STAT1, 2, 4, 5A, 5B, and 6, which are activated in a similar manner downstream of surface receptors. Binding of their ligand by these receptors, leads to the activation of receptor-associated Janus activating kinases (JAKs). The activated JAKs then phosphorylate the receptor providing docking sites for STATs, which in turn become tyrosine phosphorylated. This leads to the formation of homodimers or heterodimers, followed by translocation to the nucleus where the dimers bind to DNA and induce transcription of a broad range of target genes (1, 2).

Importantly, many of the cytokine receptors that lead to STAT3 activation are expressed by lymphocytes including those for IL-6, IL-10, IL-21, IL-23, and IFNs. The critical role of STAT3 in lymphocyte biology was highlighted by the discovery that loss-of-function (LOF) mutations in *STAT3* cause the primary immunodeficiency autosomal dominant hyper IgE syndrome (AD-HIES), which is characterized by defects in both T and B cells (3, 4). More recently, gain-of-function (GOF) mutations in *STAT3* were also identified, this time in patients who presented with early onset autoimmunity as well as immunodeficiency (5–7). These diseases demonstrate that STAT3 plays a central role in regulation of immune responses.

## Immunodeficiency Caused by *STAT3*^LOF^ Mutations

Autosomal dominant hyper IgE syndrome was first described about 50 years ago, but it was not until 2007 that two groups demonstrated that it is caused by heterozygous LOF mutations in *STAT3* (3, 4). AD-HIES is characterized by a range of immunological manifestations including elevated IgE, eczema, chronic mucocutaneous candidiasis (CMC), recurrent staphylococcal infections, and pneumonias. Patients also display non-immunological manifestations such as joint hyperextensibility, facial dysmorphism, and retention of primary teeth (8). Since the initial description over 89 disease-causing mutations in *STAT3* have been reported and are found distributed throughout the STAT3 molecule (9, 10). These mutations all lead to the same clinical phenotype, presumably because while different mutations impair signaling at different stages, they all impair the ability of STAT3 to bind to DNA and induce gene transcription (11). It should be noted that due to the dimerization step in the STAT3 signaling pathway these heterozygous mutations in *STAT3* work in a dominant negative manner. That is, in patient cells, 75% of STAT3 dimers would contain at least one LOF STAT3 molecule and thus be dysfuctional, leaving only 25% of dimers functioning normally (3, 4). Thus, AD-HIES results in severely compromised, but not completely ablated, STAT3 signaling. This 25% of residual STAT3 function is presumably critical for survival as germline deletion of *Stat3* in mice is embryonically lethal (12).

## Immune Dysregulation Caused by *STAT3*^GOF^ Mutations

More recently, patients with heterozygous GOF mutations in *STAT3* have also been described (5–7). These patients present with early onset autoimmunity and/or lymphoproliferation. The range of autoimmune manifestations is broad and includes cytopenias, type I diabetes, enteropathy, scleroderma, arthritis, and thyroid disease (5–7). However, many of these patients were also reported to suffer from recurrent or severe infections as well as hypogammaglobulinemia (6, 7) suggesting concurrent immunodeficiency. Overall, the clinical phenotype of the patients has been found to be quite variable and unaffected family members who carried STAT3^GOF^ mutations have also been identified suggesting there is incomplete disease penetrance and that other factors influence the pathogenicity of the mutations (7, 13).

The molecular mechanism that results in GOF from these germline mutations has not been extensively characterized; however, the varied patient phenotype suggests there may be more divergence in mechanism than is observed with LOF mutations. It has been observed that most disease causing GOF mutations do not alter phosphorylation; however, these mutations generally lead to increased transcriptional activity of STAT3 target genes in unstimulated and/or stimulated cells (6, 7). This in turn leads to upregulation of STAT3 target genes such as *SOCS3* (7). Interestingly, SOCS3 can regulate the activation of STAT family members, and cells from these patients were found to have reduced STAT5 phosphorylation in response to IL-2, and STAT1 phosphorylation in response to IFNγ (7). Moreover, some of the symptoms of STAT3^GOF^ patients are similar to those observed in STAT5b LOF patients (14) suggesting that reduced STAT5 activation may partially explain the phenotype (discussed below).

## The Role of STAT3 in B Cells

Multiple findings in patients with dysregulated STAT3 function point to a role for STAT3 in regulating human B cells responses. For example, although patients with STAT3^LOF^ mutations have relatively normal levels of total serum IgM, IgG, and IgA, they have elevated levels of serum IgE, defects in antigen specific antibody responses and reduced memory B cells (8, 15–19). Further, the STAT3-activating cytokines IL-21, and to a lesser extent IL-10, are potent B cell activators. In combination with CD40L, IL-21 and IL-10 are capable of inducing the proliferation, class switching, and differentiation of human B cells (19, 20). Interestingly, some, but not all, of the actions of IL-21 and IL-10 were found to be disrupted in B cells from AD-HIES patients. Specifically, STAT3^LOF^ naïve B cells were unable to differentiate into antibody secreting cells in response to CD40L and IL-21 (19, 21) and failed to upregulate key transcriptional regulators of the plasma cell program such as BLIMP-1 and XBP-1 (19, 21). In contrast, IL-21 was able to induce normal levels of switching to IgG from naïve STAT3^LOF^ B cells and could stimulate increased levels of proliferation from these cells compared to cultures with CD40L alone, albeit lower than what was observed in naive B cells from healthy controls responding to CD40L and IL-21 (19). This decreased proliferation/expansion could at least partially be attributed to an increase in cell death (19). It must be remembered, however, that these patient cells retain 25% STAT3 activity so it unclear whether the responses to IL-21 that are preserved reflect STAT3-independent effects of IL-21 or the function of the residual STAT3. Some insight can be gained from mouse models of B cell specific deletion of *Stat3* in which all STAT3 function is ablated in these cells. These models showed relatively normal switching to IgG (22, 23) but decreased expansion and/or maintenance of B cells to a T cell-dependent antigen resulting in fewer germinal center B cells (23, 24). Further STAT3 deficiency also resulted in a defect in affinity maturation (23) although cells still underwent somatic hypermutation (19, 23). Together these studies reveal that STAT3 is required in naïve B cells to induce plasma cell formation, survival, and expansion of responding B cells but not for regulating switching to IgG. Interestingly, the small number of memory cells that do emerge in patients with STAT3^LOF^ can respond normally to IL-21 to form antibody-secreting cells (21). This demonstrates that naïve and memory B cells have differential requirements for STAT3, which may have important implications for attempts to target the STAT3 pathway therapeutically.

Confirmation of the critical role of IL-21 upstream of STAT3 was provided by the identification of patients with LOF mutation in *IL21R* and *IL21*. These patients displayed similar B cell defects to those with LOF *STAT3* mutations such as reduced memory B cells, poor responses to vaccination, and elevated levels of IgE (21, 25–28). This clearly demonstrates the importance of the IL-21/STAT3 signaling axis in human B cell function. However, the exact molecular mechanism that leads to decreased memory cells and increased IgE is still unclear. Interestingly, a study of a patient with somatic mosaicism of the *STAT3* mutation has shown that STAT3^LOF^ cells did not generate memory CD4^+^ or CD8^+^ T cells but the mutation was present in memory B cells, suggesting that there was an intrinsic requirement for STAT3 in T cells but not B cells for the generation of memory (29). Thus, it may be that the decrease in memory B cells is secondary to aberrant function of other cell types such as T follicular helper (Tfh) cells (discussed below).

B cells can influence immune responses through their role in antigen presentation and the production of cytokines. IL-10 produced by B cells has been implicated in a regulatory role in immune responses (30). Interestingly, STAT3^LOF^ B cells have been shown to produce less IL-10 following stimulation, than normal controls (31) suggesting that this regulatory function of B cells may also be altered.

Given this critical role of IL-21/STAT3 in B cell differentiation, it might be predicted that STAT3^GOF^ patients would exhibit increased B cell activity. Surprisingly, however, some of these patients seem to display hallmarks of B cell dysfunction such as hypogammaglobulinemia and decreased switched memory B cells (6, 7). Conversely, many of these patients seem to display antibody-mediated autoimmunity suggesting that B cell tolerance is disturbed (5–7). Unfortunately, little functional work has been done on B cells from these patients so it remains unclear if there are B cell intrinsic effects of STAT3 over activation or whether this is secondary to defects in other cells such as Tfh cells or regulatory T cells (Tregs) (discussed below).

## The Role of STAT3 in CD4^+^ T Cells

Naive CD4^+^ T cells are able differentiate into distinct effector subsets that play specific roles in the immune response. These subsets include Th1, Th2, Th9, Th17, Th22, Tfh cells, and Tregs. The differentiation of CD4^+^ T cells is determined by the cytokine milieu at the time of activation and numerous STAT3-signaling cytokines have been implicated in this process (32, 33). Analysis of patients with *STAT3* mutations has provided key insights into the role of STAT3 in controlling these processes.

## Th17

The differentiation of human Th17 cells is controlled by the action of several STAT3-activating cytokines including IL-6, IL-21, and IL-23 (34–36). Analysis of AD-HIES patients revealed a deficiency in Th17 cells in the blood of these patients as measured by expression of CCR6 and the production of IL-17A, IL-17F, and IL-22 (28, 37–40). Furthermore, STAT3^LOF^ naïve CD4^+^ T cells from these patients fail to differentiate into IL-17-expressing cells *in vitro* (38, 41). Together, this not only demonstrates an essential requirement for STAT3 signaling in the generation of human Th17 cells but also provides an explanation for the CMC observed in AD-HIES patients as IL-17-mediated immunity is crucial for control of candida infections (42, 43). Interestingly, patients with GOF mutations in STAT1 also display defects in the generation of Th17 cells and susceptibility to candida infections demonstrating that balanced STAT1/STAT3 signaling is required for generation of these cells (41, 44, 45). On the other hand, patients with STAT3^GOF^ were not found to have increased IL-17-expressing CD4^+^ T cells suggesting that, while STAT3 is required for the generation of these cells, over activation alone is not sufficient to drive Th17 differentiation (6, 7). However, more detailed analysis of these STAT3^GOF^ CD4^+^ T cells may be required to definitively conclude this as some patients may have increased Th17 cells (46).

## Th1/Th2

In contrast to Th17 cells, the generation of human Th1 and Th2 is thought to act primarily through IL-12/STAT4 and IL-4/STAT6 signaling, respectively (32, 33). Consistent with this, generation of these populations was found to be largely STAT3-independent, as shown by normal frequencies of CXCR3^+^CCR6^−^ and CXCR3^−^CCR6^−^ and IFNγ-producing and IL-4, IL-5, IL-13-producing cells, respectively, in AD-HIES patients (28, 37, 38). Similarly, naïve CD4^+^ T cells from STAT3^LOF^ patients could differentiate into Th1 or Th2 cells when cultured under the relevant polarizing conditions (41, 47). Interestingly, IFNγ expression tended to be increased in STAT3-deficient CD4^+^ T cells (28, 41), suggesting STAT3 signaling may inhibit Th1 cell differentiation.

## Th9

Human Th9 cells develop in the presence of TGFβ and IL-4 (48, 49); however, they can also be induced by the addition of TGFβ to Th17 polarizing conditions (i.e., IL-1β/IL-6/IL-21/IL-23) (48), suggesting STAT3 may be involved in Th9 cell differentiation. Consistent with this, addition of IL-6, IL-10, or IL-21 to Th9 polarizing conditions enhanced IL-9 expression (50). In contrast, IL-27 partially suppressed TGFβ and IL-4-induced IL-9 expression (50). Since IL-6, IL-10, IL-21, and IL-27 are capable of activating both STAT1 and STAT3, further investigation is required to determine if the regulation of IL-9 production by these cytokines results from both STATs, or whether one STAT has a dominant function in regulating IL-9 production. However, one paper found that IL-9 production in responses to candida antigens was decreased in patients with STAT3^LOF^ mutations (51) suggesting that STAT3 may be important, at least under some conditions, for the induction of IL-9.

## Tfh

Like Th17 differentiation, the generation of human Tfh cells is driven by numerous STAT3-ativating cytokines, namely IL-6, IL-12, IL-21, and IL-27. Consistent with a requirement for STAT3 to induce this differentiation program, patients with STAT3^LOF^ have a reduction in circulating CXCR5^+^ Tfh cells (28, 52, 53), and naïve STAT3^LOF^ CD4^+^ T cells failed to differentiate *in vitro* into IL-21-producing Tfh-like cells (41, 52, 54). The role of STAT3 in differentiation and/or function of Tfh cells has also been demonstrated in mouse studies of Stat3-deficient T cells (55–59). However, the degree to which Stat3 is required seems to be dependent on the presence of other signals such as STAT1 and type 1 IFNs as well as the site of the immune response (57, 59, 60). This defect in Tfh cells in AD-HIES would contribute to impaired humoral immunity in these patients and potentially to the deficiency in memory B cells. *In vitro*, IL-12 was found to be the main driver of IL-21-producing cells (61, 62), but since patients with defects in IL-12R signaling do not present with impaired humoral immunity (63–65), it is likely that the other STAT3-dependent cytokines IL-6, IL-21, and/or IL-27 plays a redundant role in this process *in vivo*. Consistent with this, IL-6 and IL-21 were found to induce ICOS expression in cord blood CD4^+^ T cells in a STAT3-dependent manner (53, 66), and patients with mutations in IL21/IL21R show defects in Tfh cell development and function (28, 41).

## Regulatory T Cell

Studies in mice have suggested that IL-6/STAT3 signaling inhibits Treg differentiation inasmuch as Stat3^−/−^ CD4^+^ T cells stimulated under Th17 conditions (TGF-β^+^ IL-6 in mice) showed decreased Th17 differentiation and increased Treg differentiation (67–69). However, normal frequencies of Tregs (defined as CD25^hi^CD127^lo^ or FoxP3^+^) were reported in AD-HIES patients (28, 38, 47) and Tregs from these patients displayed normal suppressive behavior *in vitro* (47), suggesting STAT3 was somewhat redundant in controlling the generation of human Tregs. On the other hand, a recent study demonstrated that naïve CD4^+^ T cells from STAT3^LOF^ patients showed an increased propensity to develop into iTregs in culture (70). CD4^+^ T cells from these patients have also been reported to display a defect in IL-10 production (28, 38, 41) and DCs from STAT3-deficient patients failed to induce Tregs from naïve CD4^+^ T cells (47). Thus, it may be that in these patients the increased propensity of STAT3^LOF^ CD4^+^ to form induced Tregs is compensated for by the reduced ability of STAT3^LOF^ dendritic cells to induce them.

Indeed, patients with STAT3^GOF^ mutations much more clearly demonstrate a role for STAT3 in the regulation of Tregs. As discussed above, these patients display early onset autoimmunity that is reminiscent of patients with Immune dysregulation, polyendocrinopathy, enteropathy, X-linked (IPEX) syndrome, which is caused by mutations in *FoxP3* leading to a loss of Treg function (71). These overlapping clinical phenotypes suggested that STAT3^GOF^ patients also have dysfunctional Tregs. Consistent with this, they were found to have lower percentage of FoxP3^+^ cells in their blood and lower CD25 expression on their Tregs (7). This is thought to be due to increased SOCS3 levels that inhibit the activation of STAT5 downstream of IL-2 (7).

Studies in mice have now demonstrated that there are different populations of Tregs that seem to be specialized for inhibiting particular T-helper populations (72). Thus, a population of Treg cells that express CCR6 and are specialized for suppressing Th17 cells has been described. These “Treg17” cells, like the Th17 cells they suppress, were found to be dependent on STAT3 signaling (73). AD-HIES patients were also shown to have decreased CCR6^+^ Tregs suggesting that human “Treg17” cells may also exist and be dependent on STAT3 (74).

Taken together, these data suggest STAT3 plays a complex role in the regulation of Treg responses and care should be taken in targeting this pathway as a means of regulating Treg responses.

## The Role of STAT3 in CD8^+^ T Cells

STAT3 activating cytokines such as IL-21 also play a role in regulating CD8^+^ T cells. Studies on STAT3^LOF^ CD8^+^ T cells showed they had impaired induction of perforin and granzyme B in response to IL-21; however, this could be rescued by strong TCR ligation (75). In contrast, proliferation induced by IL-21 was not affected in naïve STAT3^LOF^ CD8^+^ T cells (75). STAT3 deficiency, however, did result in reduced memory CD8^+^ T cells (29, 75), an effect that was shown to be cell intrinsic (29). IL-21R patients also showed decreases in memory CD8^+^ T cell populations suggesting that IL-21 may be a cytokine upstream of STAT3 that contributes to the maintenance of memory cells (75). Patients with AD-HIES also show increased susceptibility to reactivation of viruses such as EBV and VZV (29) indicating that STAT3^LOF^ CD8^+^ T cells may be defective in their ability to control these chronic infections.

## STAT3 in Autoimmunity

**Figure 1:**
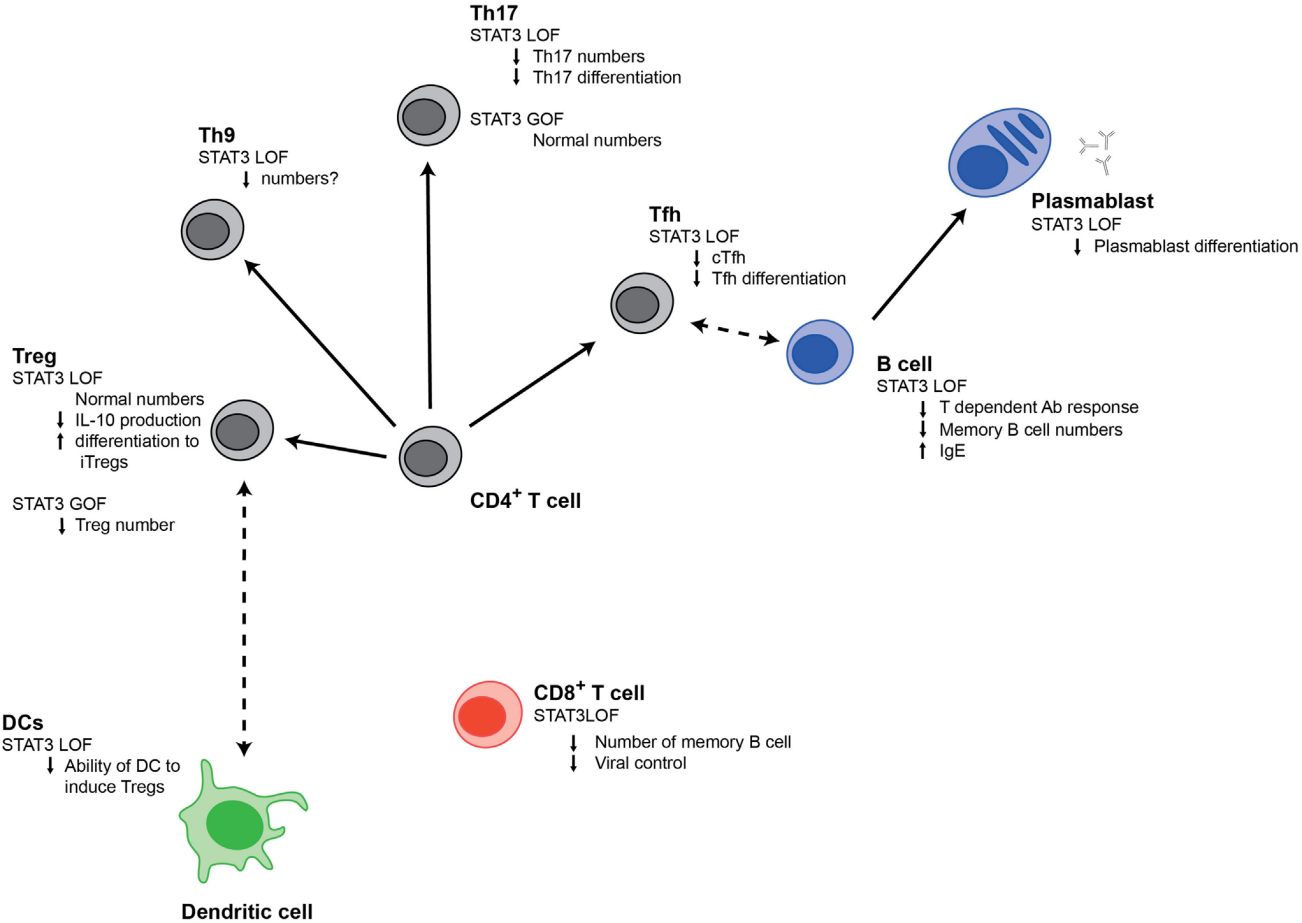
Signal transducer and activator of transcription 3 (STAT3) regulates multiple populations of immune cells. Changes in different immune cell populations that are observed in STAT3^LOF^ or STAT3^GOF^ patients are shown.

STAT3^GOF^ patients provide a clear demonstration that STAT3 plays an important role in controlling autoimmunity. However, previous evidence from other disease states had already indicated that STAT3 played an important role in the regulation of autoimmunity. In particular, multiple studies have associated polymorphisms in *STAT3* with various autoimmune conditions including Crohn’s disease, ulcerative colitis, psoriasis, and Behcet’s disease (76–78). Furthermore, many of the cell populations that STAT3 can induce (Figure 1) have been implicated in driving autoimmunity; this includes Tfh cells and B cells, which support autoantibody production and Th9 and Th17 cells, can produce potentially damaging cytokines (79, 80). Conversely, STAT3 can inhibit Tregs, which act to restrain destructive immune responses.

Interestingly, somatic STAT3^GOF^ mutations have been reported in large granular lymphocytic (LGL) leukemia (81, 82) and are associated with higher rates of autoimmune complications such as rheumatoid arthritis and autoimmune cytopenias (81–83). As these LGL leukemias are of either CD8^+^ T cell or NK cell origin, this suggests that STAT3 overactivation in CD8^+^ T cells or NK cells alone may be sufficient to drive autoimmunity, possibly by inducing the production of cytokines/inflammatory mediators (84).

Taken together, these data are consistent with a role for STAT3 in promoting autoimmunity; however, STAT3 is also likely to play a role in inhibiting damaging responses downstream of IL-10. Indeed, germline LOF mutations in the genes coding for IL-10 or IL-10R are a major cause of early onset inflammatory bowel disease (85), demonstrating a critical requirement for IL-10 signaling (presumably through STAT3) to maintain tolerance in the bowel.

It remains unclear which of these many roles of STAT3 in multiple cell types underlies the phenotype in STAT3^GOF^ patients. Certainly, the similarities with IPEX (71, 86) point to a clear role of Tregs in the phenotype; however, contributions from other pathways are also likely to make contributions. Further research will help clarify this and in turn pave the way for targeted treatments for both rare patients with STAT3^GOF^ mutations as well as patients who suffer from autoimmunity more generally.

## Author Contributions

ED, SP, AK, and CM wrote and edited the manuscript.

## Conflict of Interest Statement

The authors declare that the research was conducted in the absence of any commercial or financial relationships that could be construed as a potential conflict of interest.
